# New Approach for Processing Recycled Carbon Staple Fiber Yarns into Unidirectionally Reinforced Recycled Carbon Staple Fiber Tape

**DOI:** 10.3390/polym15234575

**Published:** 2023-11-30

**Authors:** Martin Detzel, Peter Mitschang, Ulf Breuer

**Affiliations:** Leibniz-Institut fuer Verbundwerkstoffe GmbH, Erwin-Schroedinger-Strasse 58, 67663 Kaiserslautern, Germany; peter.mitschang@leibniz-ivw.de (P.M.); ulf.breuer@leibniz-ivw.de (U.B.)

**Keywords:** recycled carbon fibers, staple fiber yarn, tape manufacturing, thermoplastic composite, alignment of discontinuous fiber, mechanical properties

## Abstract

This study describes a novel process in which staple fiber yarns made from recycled carbon fibers (rCFs) and polyamide 6 (PA6) fibers are further processed into semi-finished tape products in a modified impregnation and calendaring process. In this process, the staple fiber yarns are heated above the melting temperature of the polymer, impregnated, and stretched to staple fiber tapes (SF tapes) in the calendaring unit. SF tapes with different degrees of stretching and/or repasses were produced. The individual width and thickness were measured in line by a laser profile sensor. From these tapes, preforms were manually laid and processed into laminates in an autoclave. The important physical properties of the unidirectionally reinforced laminates made of the tapes were compared with organic sheets wound from staple fiber yarns. With increasing stretching, both the fiber orientation and mechanical properties improved compared to the organic sheets made from unstretched staple fiber yarns. An improvement in fiber orientation relative to the process direction from 66.3% to 91.9% (between ±10°) and 39.1% to 71.6% (between ±5°), respectively, was achieved for a two-stage stretched tape. The tensile and flexural moduli were increased by 15.2% and 14.5%, respectively.

## 1. Introduction

With the increased use of carbon fiber-reinforced polymer composites (CFRPCs) in the transportation sector and aircraft, the generation of CFRPC waste is also growing [1,2,3]. In 2021, the global demand for CF was about 92.0 kt, with a forecasted demand of about 122 kt by 2024 [4]. During the production of CFRPCs, approximately 40% of the global CFRPC waste is generated, with fabric cut-offs accounting for more than 60% of the total [1,5,6]. The CFRPC waste can be classified into three classes: process waste (1—dry fibers, such as leftover spools or fabric scraps, 2—expired prepregs and partially cured semi-finished products), and 3—end-of-life (EoL) waste [2,6,7,8]. Since waste from Class 1 retains its original sizing, it can be easily reintegrated into the value chain without significant effort. Typically, the fibers are only cut to a uniform length [6,7].

For waste from Classes 2 and 3, the recovery of rCF involves the initial removal of the matrix through thermal or chemical processes. In order to recycle EoL waste, there are also mechanical options, such as shredding or grinding. These methods result in significant fiber shortening, limiting the recycled material to applications with low mechanical requirements. Due to this “downcycling” effect, mechanical methods are of minor interest. The target should be to obtain fibers that are as long as possible in order to achieve good mechanical properties in high-value second-life parts [1,2,5,7,9,10].

Thermal recycling processes apply high temperatures to remove the matrix and recover the fibers. These thermal methods include pyrolysis [10] and the fluidized bed process [2]. On the other hand, chemical methods utilize solvents, both supercritical and subcritical, to dissolve the matrix into its constituents. In addition to these established methods, there are several other techniques, such as electrochemical processes like high-voltage fragmentation, electrohydraulic fragmentation, and electrically driven heterocatalytic decomposition [2,9]. In these methods, high currents are utilized to break down the CFRPC to its constituents. However, these methods are limited by their high-energy input, with a significant portion of the energy not being effectively utilized in the recycled material [1,2,7,9,11].

Both thermal and chemical processes yield fibers with residue-free surfaces under optimal process conditions. Depending on the recycling method, the original sizing of the fibers can be preserved. While thermal methods often lead to the complete removal of the sizing, the superheated steam process can retain the sizing of the fibers [1]. After the recycling process, the rCFs obtained from Classes 2 and 3 may need to be coated with a new sizing, depending on their further use.

In 2015, Oliveux et al. [9] described the energy-saving potential of the use of pyrolized fibers. Virgin fibers consume approximately 198–598 MJ/kg during production, while the rCFs obtained from the pyrolysis process consume only 10.3–35.7 MJ/kg of energy. In addition to the energy-saving potential, a significant part of the costs can be saved by using pyrolized fibers. They estimated the cost of newly produced CF at USD 33–66/kg and rCF at USD 13–19/kg.

In a 2022 study by Kooduvalli et al. [3], the life cycle assessment for CFRPC recycling was investigated. Different pyrolysis and solvolysis processes were evaluated. The most common process used in the industry is pyrolysis, where the gas produced (other than oil) is used as fuel for the furnace. An electrically operated furnace needs approximately 115.9 MJ energy per kg of CFRPC to remove the matrix. When using natural gas, it reduces the consumption to approximately 41.3 MJ/kg. By utilizing pyrolysis oil, the energy consumption can be further reduced to 81.6 MJ/kg for electrically operated furnaces and 7.04 MJ/kg for those using natural gas. The energy consumption during solvolysis is mainly influenced by the used solvent. Among the solvolysis processes considered in the publication, the lowest energy consumption was calculated for solvolysis using deionized water as solvent (256.8 MJ/kg), and, in contrast to this, the highest energy consumption was observed when using a mixture of acetone and water (277.5 MJ/kg). The study shows that, in comparison to virgin fibers, both the use of rCF fibers from solvolysis and from pyrolysis can result in energy savings. Furthermore, pyrolysis is significantly less energy-intensive compared to solvolysis.

After the fiber recovery process, there are several options for processing the fibers, such as by injection molding using short fibers and sheet-molding compounds (SMCs) or bulk-molding compounds (BMCs) using short and medium length fibers with no specific fiber orientation [2,5,6,8,9]. Additionally, isotropic textiles, like meshes and mats, can be produced by air-laying or wet-laying processes [12,13,14,15].

The highest potential for harnessing the mechanical performance of rCFs regards methods where the fibers are aligned. In the wet-laying processes, the rCFs or a combination of rCFs and thermoplastic fibers are mixed with a solution to ensure a uniform dispersion of the fibers. In order to filter the fiber from the suspension, the mixture is pumped onto a sieve. Subsequently, the remaining fiber mats are dried and, optionally, provided with a binder. It is possible to set the fiber orientation by using a moving sieve and/or the flow geometry of the headbox [7,16].

In the carding process, continuous nonwovens are produced by the mechanical opening and blending of fiber bundles. However, during carding, there is a shortening of the rCFs. Initially, fiber bales are opened and disentangled to fiber tufts by using a bale breaker. During the opening phase, these fiber tufts are further opened to individual fibers and thoroughly mixed. For the production of hybrid nonwovens, thermoplastic fibers can be added to this phase to enhance fiber-fiber cohesion, thereby improving the orientation of the rCFs during carding [2,17]. In the carding process, the opened fibers are fed to toothed cylinders. The fibers are taken off the main cylinder (tambour or swift), disentangled, and thoroughly mixed by worker and stripper pairs. Subsequently, the fibers are fed back onto the main cylinder. After the carded web has passed through several pairs of workers and strippers, it is removed from the main cylinder by a roller (doofer). By increasing the number of worker and stripper pairs, the fiber orientation can be further improved, but at the expense of fiber length [2,6,7,13,17].

By stretching in one direction, the carded nonwoven webs can be converted into slivers [2,18,19]. These slivers can be further processed to yarns by additional stretching to achieve the desired fineness and consolidation [18,19]. The consolidation can be obtained through twisting, wrapping the fiber bundle, or enveloping it with sheath fibers, depending on the yarn formation process [20,21,22,23,24,25,26]. These yarns can be directly used for the production of components or first processed into fabrics and noncrimp fabrics [23,26,27].

Numerous studies focus on the production of rCF semi-finished products in tape form. Rimmel et al. [28] used slivers made from pyrolized rCFs, with fiber lengths ranging from 70–120 mm. A thermoplastic binder band was folded around these slivers, then thermally activated and consolidated into a binder tape. These binder tapes were further utilized in a tape-laying process to semi-consolidated tape preforms. Subsequently, these preforms were processed into composite materials by taking two process routes. In the thermoplastic route, the preforms with a high thermoplastic binder content were directly consolidated to laminates, whereas, in the thermosetting route, a preform with a low thermoplastic binder content was impregnated by using an RTM process.

At the University of Bordeaux, a method was developed to produce continuous tapes with unidirectional fiber orientation containing fiber lengths ranging from 40 to 260 mm. The fibers obtained from recycled woven fabrics were aligned by a moving U-shaped rail. A thermoplastic binder was applied to the fibers and then melted by infrared heating. The subsequent consolidation using rollers to form tapes 25 and 50 mm in width was achieved. These tapes reached a maximum fiber alignment of 91% within 0° ± 10° of the production direction. Subsequently, these tapes were further processed in compression molding with an epoxy resin [29,30,31,32].

In the HiPerDiF (high-performance discontinuous fiber) method, which was developed at the University of Bristol, short fibers (1–6 mm) are used for tape production. The fibers are first dispersed in a liquid-dispersing medium. By accelerating the dispersing medium through a nozzle, the fibers are partially aligned. By spraying the dispersion onto a plate, the fibers align perpendicular to the direction of the jet and fall onto a moving perforated belt while maintaining their orientation. Then, the liquid is removed through a suction system. Subsequently, the fibers are dried and impregnated with a matrix. Different fibers, as well as fiber blends, can be used in this process and can be impregnated with a thermoplastic and thermoset matrix, as well as vitrimers. For the composites produced with a fiber volume content of 41%, 65% of the fibers were aligned within ±3°, and for the composites produced with a fiber volume content of 55%, 67% of the fibers were aligned within ±3° [33,34,35,36,37,38,39,40,41,42,43,44].

In the tailored universal feedstock for forming (TuFF) manufacturing processes, fibers are dispersed in a liquid reservoir and then distributed on a moving porous conveyor belt. The liquid is suctioned through the porous belt via a vacuum, and the highly aligned fibers are stabilized. This allows for the creation of unidirectional aligned nonwovens from dry fibers with controlled areal weights of ~8 g/m² using various fiber materials (CF, GF, NF, etc.) or fiber combinations. These dry nonwovens can be further processed in either a thermoplastic or thermosetting route. In studies, carbon fibers (IM7) were cut to lengths of 1–7 mm and processed to prepregs and coupons by using this method. It was observed that longer fiber lengths result in poorer fiber orientation. For a laminate with a fiber length of 3 mm, 98.4% of the fibers were aligned within ±10°, and 94% of the fibers were aligned within ±5° [45]. In additional investigations, the deformation limits of the TuFF material were examined with respect to strain rate and strain deformation at temperatures of 280, 300, 310, 320, and 330 °C. It was observed that the material exhibits lower levels of strain at high strain rates, leading to premature failure. By reducing the strain rate, higher levels of strain could be achieved, as the material had sufficient time to relax [45,46,47,48,49,50].

In the research conducted at TU Dresden, uniform, highly aligned hybrid slivers were further processed into tapes. Multiple slivers were arranged in parallel, stretched, and heated above the polymer melting temperature with an infrared heater. Subsequently, the tapes were consolidated into a uniform thickness by applying pressure using two consolidation rollers. Tapes with fiber lengths ranging from 40 to 100 mm were produced at a processing speed of 10 m/min [51,52,53].

Akonda et al. also processed slivers made into tapes made from PET and rCF (fiber length 60 mm). Thermal consolidation was carried out at 240 °C, and tapes with a thickness of 0.5 mm and a width of 180 mm were produced from 10 slivers. For further processing, these wide tapes were slit to a width of 20 mm and woven [54].

Staple fiber tapes based on slivers were also produced at the German Institutes for Textile and Fiber Research (DITF). rCF staple fibers with a length of 80 mm from production waste were used. The slivers made of PA6 and rCF were heated above the melting temperature of the polymer with hot air blowers and then consolidated between 80 °C using warm consolidation rollers. Other heating methods for thermoplastic fibers, such as laser, ultrasound, hot calendar, and heated pultrusion molding, were also investigated. However, due to the short duration required for energy transfer, it was shown that the hot air blower is the most suitable option. Tapes with average widths of 13–13.5 mm were manufactured. They observed that when the process temperature increases, the tape thickness decreases because higher temperatures allow for better compaction and consolidation of the tape [55].

In the project “Mai RecyTape”, conducted by the Institute of Textile Technology at RWTH Aachen University, carded webs made of PET and rCF were directly processed into tapes. The carded webs consisted of 80% rCF fibers with an average length of 48.8 mm and 20% PET fibers. These carded webs were further processed in a calendaring unit at Freudenberg Haushaltsprodukte Augsburg GmbH. The resulting tape was pressed using a thermoplastic route to a laminate structure with additional thermoplastic nonwovens. When using the thermoset route, these tapes were processed by using the vacuum resin infusion process with epoxy resin. For a demonstrator fabric, 25 mm wide nonwoven tapes were slit and woven with glass fiber tape [56].

According to the author’s knowledge, the current manufacturing methods that are capable of achieving high fiber orientations are not suitable for long carbon fibers (>50 mm). This results in a reduction in performance, and the potential of long rCFs in these tapes is not fully exploited. For this reason, this study describes a novel process approach for further processing staple fiber yarns made of long rCFs and PA6, with the aim of enhancing fiber orientation and producing staple fiber tapes. The improved fiber alignment of the long rCFs, in contrast to the previously mentioned studies, will increase the potential of long rCFs. For this, the input materials, as well as the manufactured tapes, were characterized and examined. Furthermore, fiber orientation measurements and mechanical tests were conducted on a laminate wound from the staple fiber yarns and tape-laid laminates. After the mechanical investigations, the influence of the tape manufacturing process on the thermal properties was examined. From these results, the parameters for optimizing the manufacturing process and future research priorities for achieving higher fiber alignment and improved mechanical properties were derived.

## 2. Materials and Methods

### 2.1. Staple Fiber Yarn

The staple fiber yarns were produced in the wrap spinning process by Wagenfelder Spinnerei GmbH (Wagenfeld, Germany). According to the manufacturer, the staple fiber yarns consist of 60 wt% CF staple fibers and 40 wt% PA6 staple fibers with a titer of 500 tex. The CFs of the manufacturer Zoltek (St. Louis, MO, USA) for the type Panex^®^ 35 were used. The fibers were obtained from spool remnants and were cut to a length of 80 mm. No pyrolysis or solvolysis was applied, which leads to the benefit of the original sizing still being present in the fibers. Some important physical properties of the fibers are listed in Table 1.

**Figure 1:**
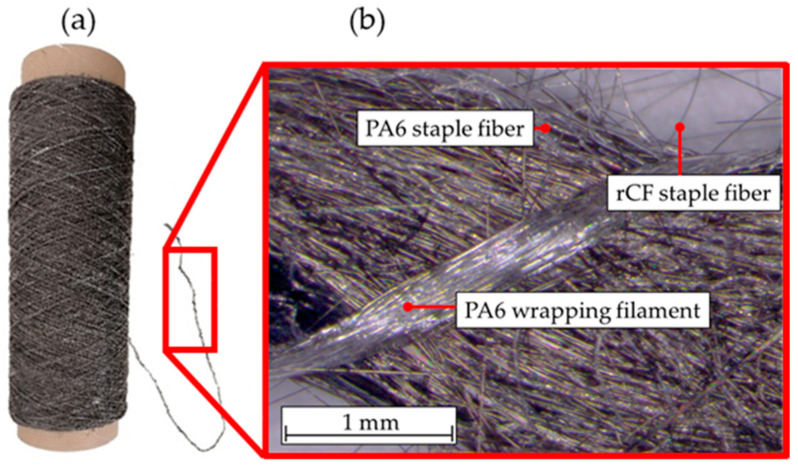
(**a**) rCF yarn and (**b**) close-up of the yarn.

Figure 1 shows a spool of the rCF staple fiber yarn and a close-up of the yarn with the respective components. PA6 staple fiber with a length between 75 and 110 mm and a fineness of 6.7 dtex was used as the thermoplastic matrix, together with a wrapping filament made of another PA6. Due to processability, two different PA6 types had to be chosen. Due to the wrap spinning process, an undulation is induced in the staple fiber yarn, which leads to a misalignment of the fibers. This misalignment causes a decrease in the mechanical properties, as the fibers are no longer perfectly aligned in the load direction [27].

### 2.2. Tape Manufacturing

**Figure 2:**
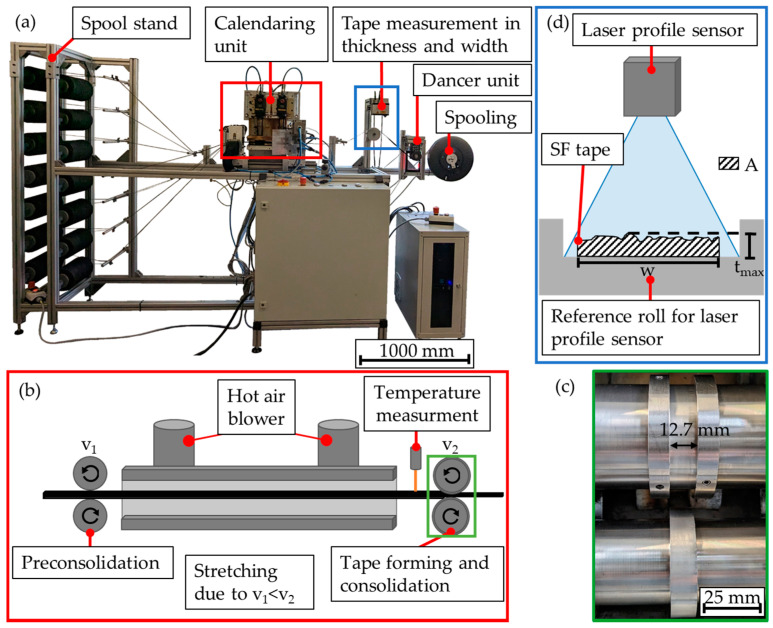
(**a**) Tape production line, highlighting the most important components; (**b**) schematic illustration of the calendaring process; (**c**) close-up of the consolidation rolls, and (**d**) schematic illustration of the measurement setup of the laser profile sensor.

Tape production was carried out on the tape production line shown in Figure 2a. The process can be regarded as a modified impregnation and calendaring process. The staple fiber yarn spools were stored in a mechanically braked spool stand. These staple fiber yarns were pulled off the spools by the preconsolidation rolls of the calendaring unit and were parallelized over several deflection stages before entering the calendar unit. Within the 25 cm long heating section of the calendaring unit, the staple fiber yarns were heated by two separately controllable hot air blowers (Leister LHS 21S SYSTEM, Kaegiswil, Switzerland, 230 V, 2000 W, maximum temperature 650 °C) above the melting temperature of the PA6 (see Figure 2b). A continuously adjustable speed difference (stretching factor) between the preconsolidation rolls and the consolidation rolls stretches the staple fiber yarns. This stretching allows the carbon fibers to slide off each other, which causes an alignment of the fibers in the process direction and, thus, leads to improved fiber orientation.

The consolidation rolls, which operate on the tongue-and-groove principle, consolidate the staple fiber yarns in a 12.7 mm wide groove into SF tape (see Figure 2c). The consolidation force of the rolls was applied by a pneumatic cylinder and can be continuously adjusted up to 320 N. In order to prevent excessive heating of the consolidation rolls, they were cooled with a vortex cooling system. In a vortex cooling system, the incoming compressed air is mechanically separated into warm and cold air streams. The cold air stream was used to cool the rolls, while the warm air stream was used to operate the hot air blowers. After consolidation, the SF tape was deflected again and tensioned over a roll. This roll served as a reference point for a laser profile sensor (scanCONTROL 3012-25/BL Micro-Epsilon, Ortenburg, Germany), which measured the width and thickness of the SF tape (see Figure 2d). The scanning rate of the laser profile sensor can be adjusted in 5 Hz steps from 25 Hz to 2 kHz. Depending on the measuring range, the laser profile sensor offers a lateral resolution of a min. of 26 µm to a max. of 22 µm and a height resolution of 2 µm. For the measurement of width and thickness at constant intervals, a constant winding speed of the SF tape was necessary. This was achieved by using a dancer unit, as well as a slip clutch within the spooling unit. A sensor was attached to the dancer unit to control the speed of the spooling unit so that tension was applied to the tape. After the measurement, the SF tape was wound onto Häfner spools.

In order to achieve constant vortex cooling and heating, the vortex cooling and the hot air blowers were switched on 15 and 5 min before tape production, respectively. During tape production, the temperature of the hot air blowers was set to 550 °C, and the consolidation force of the pneumatic cylinder was set to 120 N. The temperature of the staple fiber yarns and, respectively, the tape was measured by a laser pyrometer immediately after the heating zone and before tape consolidation and ranged from 280–310 °C with the set process parameters. The speed of the preconsolidation rolls (v_1_) was applied as a process variable, and the speed of the consolidation rolls (v_2_) resulted from the set speed of the preconsolidation rolls (v_1_) and the stretching factor. SF tapes were produced with a total stretching of 10% and 20%. This stretching was introduced to the SF tape either in a single pass (P1_1 and P4_1) or in two successive passes (P2_2, P3_2, and P5_2). All the parameter sets for SF tape production are listed in Table 2. The results are abbreviated according to the parameter sets and the passes in the following figures. For example, P3_2 represents the tape after a second pass through the tape manufacturing line, where the tape was stretched during the first pass by 10% and by 9% during the second pass. In addition, the total stretching factor of the tape is also shown in the following figures.

The scanning rate of the laser profile sensor depends on the speed of the consolidation rolls (v_2_) in order to achieve identical measurement distances for the tape for all test series.

### 2.3. Manufacturing of Staple Fiber Organic Sheets

**Figure 3:**
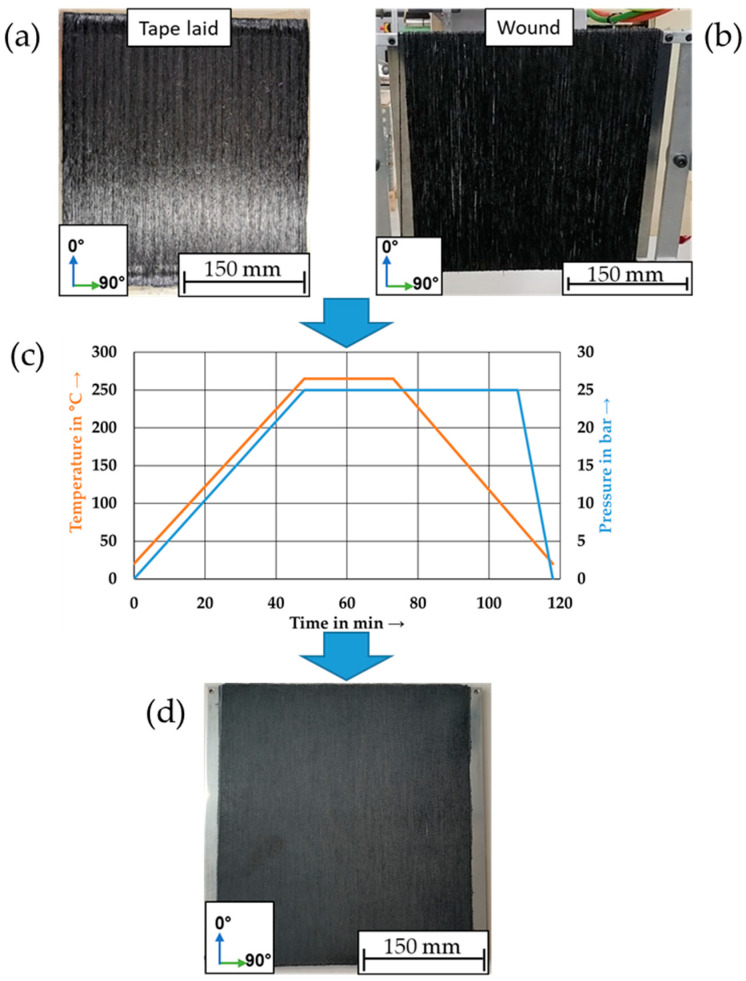
(**a**) Tape-laid laminate, (**b**) wound laminate, (**c**) autoclave process, and (**d**) consolidated laminate.

Organic sheets were produced from the previously manufactured SF tapes due to the statistical variations in the rCF starting material at the micro level; a reliable mechanical characterization was only possible at the laminate level. Thus, a mechanical characterization of the SF tapes was deliberately omitted. The unidirectional reinforced laminates were manufactured by stacking five layers of SF tapes, resulting in a laminate with a size of 300 × 300 mm^2^. The SF tapes were laid down by hand. For positioning, the tapes were tacked to each other with the aid of a soldering iron. A tape-laid preform is shown in Figure 3a.

In order to benchmark the tape production process, the reference laminates were produced by using staple fiber yarns and a winding process. The results of the reference plates are shown in the following figures abbreviated with R. For this purpose, the dry staple fiber yarns were nearly unidirectional (±0.34°) and were wound with a pulling force of 5 N onto a metal plate with dimensions of 300 × 265 mm^2^ on a winding machine from the manufacturer Roth Hydraulics GmbH. These laminates were made of eight individual layers (see Figure 3b).

The tape-laid and the wound laminates were placed between two steel plates and processed with the same autoclave cycle (see Figure 3c).

### 2.4. Characterization of the Staple Fiber Yarn and the Manufactured SF Tape

In order to determine the yarn titer, yarns with a test length (*l*) of 1 m each were cut and weighed (*m*). Equation (1) was used to determine the yarn linear density, and Equation (2) was used to determine the yarn uniformity variation coefficient (*CV_m_*).

(1) $$T_{t}=\frac{m}{l}$$

(2) $${CV}_{m}=\frac{\sqrt{\frac{\sum_{i=1}^{n}\left(T_{t_{i}-}\bar{T_{t}}\right)}{n}}}{\bar{T_{t}}}\cdot 100\%$$

The homogeneity of the produced SF tape is directly related to the homogeneity of the yarn. For the assessment of SF tape uniformity, the width and average thickness of the produced tapes were determined by using the laser profile sensor of the tape production line. The average tape thickness ($\overset{-}{t}$) was calculated by dividing the tape area (*A*) by the tape width (*w*), as direct thickness measurement using the sensor provides a maximum value as the thickness (t_max_) (see Figure 2c). This maximum value was more sensitive to errors than the averaged thickness.

(3) $$\bar{t}=\frac{A}{w}$$

### 2.5. Characterization of the Thermal Properties

The thermal properties of the two PA6 fibers, as well as the produced laminates, were investigated by thermogravimetric analysis (TGA) and differential scanning calorimetry (DSC). DSC was used to determine the melting temperature of the PA6 matrix. The analyses were performed in a DSC3+ (Mettler Toledo, Colombus, OH, USA) at a heating rate of 10 °C/min from 20 °C to 280 °C under a nitrogen supply of 30.0 mL/min.

TGA was used to determine the decomposition temperature of the PA6. The TGA experiments were conducted in a TGA (Mettler Toledo) with an air supply at a heating rate of 10 °C/min in a temperature range of 30–520 °C.

### 2.6. Determination of Fiber Orientation

A polarization camera (VCXU-50MP from Baumer GmbH, Frauenfeld, Switzerland) was used for the ex situ measurement of the fiber orientation of the laminates produced in the autoclave. The camera was combined with an HF16HA-1B lens from the manufacturer Fujifilm Corporation. Due to the limited resolution of the polarization camera, individual fibers could not be resolved; thus, the fiber orientation values were averaged within a pixel. However, this was sufficient for a qualitative comparison between the manufactured laminates. The camera was fixed to a frame to ensure a constant distance to the sample, and during the measurement, the sample was exposed to a ring light for uniform light conditions during testing. The test setup is described and shown in more detail in [57]. The software of the polarization camera captured the images in raw data form and calculated the intensity, degree of linear polarization, and the angle of polarization of the images from them. The captured images were analyzed with the software ImageJ 1.53t. The fiber orientation of the front and back sides of each laminate was determined, and then the average value was calculated.

### 2.7. Determination of the Fiber Volume Content

The fiber volume content (FVC) of the staple fiber yarn and of the manufactured laminate was determined in accordance with DIN EN ISO 1172 [58]. Three samples were taken per laminate, and due to the large deviation between the staple fiber yarns, the samples were taken and tested from 10 different spools. All samples were in the range between 5–6 g. The samples were first dried for 72 h at 80 °C and weighed (*m_Composite_*). Subsequently, the samples were heated to 450 °C for 2 h in a muffle furnace (Nabertherm GmbH type LT15/12, Lilienthal, Germany) and kept at this temperature for 4 h in order to completely degrade the PA6 matrix. The remaining CFs were then weighed (*m_rCF_*), and the fiber volume content, *v_F_*, was calculated based on the known densities of the CF and PA6, *ρ_rCF_* = 1.81 g/cm^3^ and *ρ_PA_*_6_ = 1.14 g/cm^3^, and the fiber mass ratio, m_F_, by using Equations (3) and (4).

(4) $$m_{F}=\frac{m_{rCF}}{m_{Composite}}$$

(5) $$v_{F}=\frac{1}{1+\frac{1-m_{F}}{m_{F}}\cdot \frac{\rho_{rCF}}{\rho_{PA6}}}$$

### 2.8. Mechanical Testing

#### 2.8.1. Tensile Testing

The tensile properties of the specimens in the 0° direction were tested according to DIN EN ISO 527-5 [59] on a Zwick 1474 universal testing machine (Zwick GmbH & Co. KG). The dimensions of the specimens, including load application elements, were 250 mm × 15 mm. The thickness of the laminates varied for the different stretching factors and ranged from 1.6 to 1.8 mm. A test speed of 2 mm/min and a free test length of 150 mm were used for the tensile test. The material strain was recorded by a macro displacement sensor. At least five specimens were tested for each variant. Before testing, the specimens were dried at 80 °C for 24 h, and then each specimen was measured individually, using the average of three measurements (top, middle, and bottom) to determine the width and thickness of the specimen.

#### 2.8.2. Flexural Testing

The flexural properties of the specimens were determined in the 0° direction by using a three-point bending method on a Zwick 1445 universal testing machine (Zwick GmbH & Co. KG, Ennepetal, Germany) in accordance with DIN EN ISO 14125 [60]. The specimens had a width of 15 mm, and the specimen length and the support distance used were selected according to material Class 4. A test speed of 5 mm/min was used for the flexural test. The deflection of the specimen was determined by the corrected crosshead displacement. At least five specimens were tested for each variant. Before testing, the specimens were dried at 80 °C for 24 h, and then each specimen was measured individually, using the average of three measurements (top, middle, and bottom) to determine the width and thickness of the specimen.

### 2.9. Inspection of the Fracture Surface of the Composite Test Specimens

After the tensile and flexural testing of the test specimens, the fracture surface was examined with a scanning electron microscope with EDX (SEM) Supra 40 VP (Carl Zeiss Mikroskopie GmbH, Oberkochen, Germany).

## 3. Results

### 3.1. Characterization of the Staple Fiber Yarn

**Figure 4:**
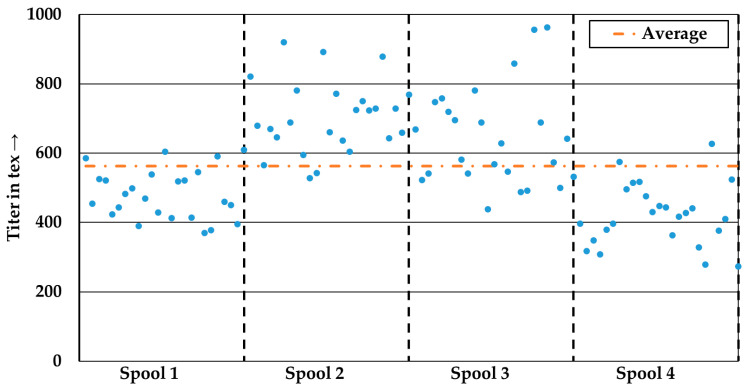
Distribution of the titer.

Figure 4 shows the titer distribution of 100 measured yarns. This yarn was extracted from four spools, resulting in 25 measured values corresponding to the yarns from a single spool. The average titer was 562 ± 155 tex, which correlates with the manufacturer’s specifications. Significant variations are observed between the individual spools, as well as within the measurements from a single spool. Spools 2 and 3 tend to have a higher titer compared to the manufacturer’s specification, whereas spools 1 and 4 tended to have a lower titer. The yarns exhibit low yarn uniformity, with a coefficient of variation (CVm) of 27.6%.

**Figure 5:**
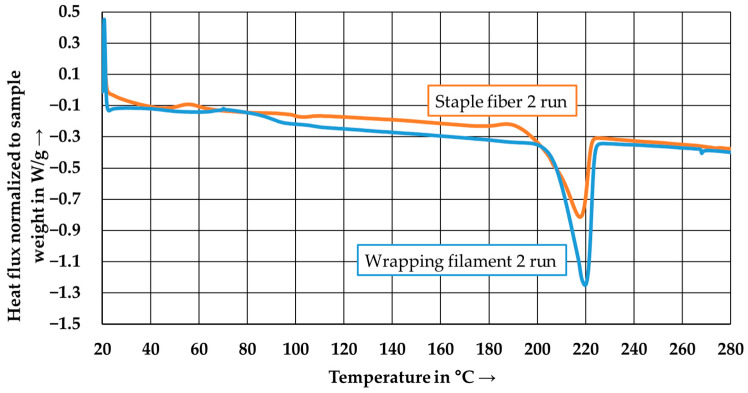
DSC-thermogram of the PA6 staple fiber and the PA6 wrapping filament.

Figure 5 shows the results of the DSC measurements conducted on the two types of PA6 used in this study. The graph represents the second heating cycle to ensure that both PA6 types were influenced by the same temperature profile (including heating and cooling). The peak melting temperature of the PA6 staple fibers in the second heating cycle is 217.6 °C, and the PA6 wrapping filament is 219.7 °C.

**Figure 6:**
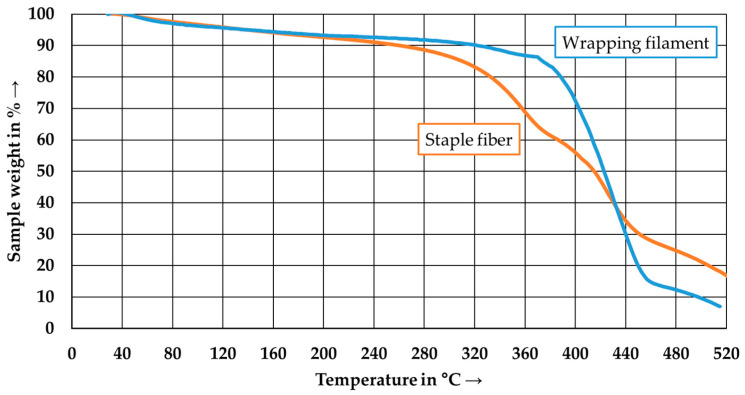
TGA-thermogram of the PA6 staple fiber and the PA6 wrapping filament.

Figure 6 presents the results of the TGA measurements conducted on the two types of PA6 used. In contrast to the results of the DSC measurements, the PA6 types exhibit different behavior. The onset temperature of the PA6 wrapping filament is approximately 385 °C, followed by rapid degradation until 460 °C. The onset temperature of the PA6 staple fiber starts at approximately 330 °C, with a two-stage degradation process. The second degradation process begins at around 420 °C and ends in the range of 460 °C.

Both the results of the DSC analysis and the findings from the TGA experiments constrain the processing parameters within the calendaring process. The polymer needs to be in a molten state during the consolidation phase, and any thermal damage to the polymer must be prevented during the process.

### 3.2. Characterization of the Staple Fiber Tape

**Figure 7:**
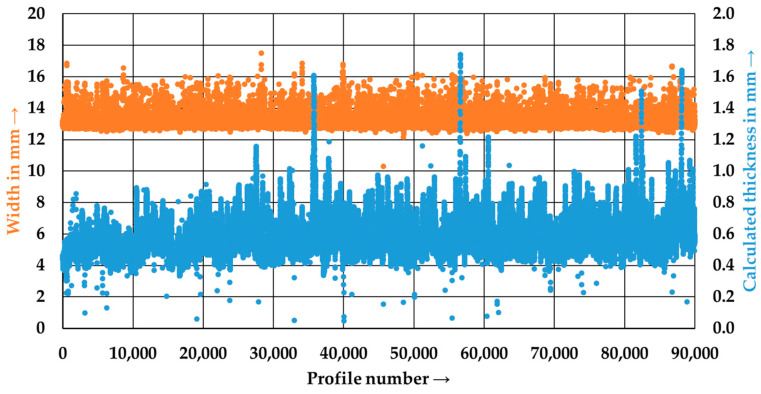
Width and calculated thickness of the tape P5_1 after the first pass through the tape manufacturing line.

In Figure 7, the typically measured width and calculated thickness of a tape (here, P5) is shown after the first (P5_1) and second pass (P5_2) through the tape manufacturing line. Each profile number represents a measurement of the tape at a distance of about 0.5 mm. In the first pass, the tape width exhibits a distinct lower limit imposed by the groove width of 12.7 mm. The mean tape width after the first pass is 13.05 ± 0.33 mm. The data points exceeding the groove width of 12.7 mm were caused by dry fibers at the edge of the tape protruding from the tape edge and detected by the sensor. The calculated tape thickness shows an average thickness of 0.58 ± 0.11 mm. The large variation can be attributed to the variation in the staple fiber yarns as well as insufficient consolidation during the first pass of the process.

**Figure 8:**
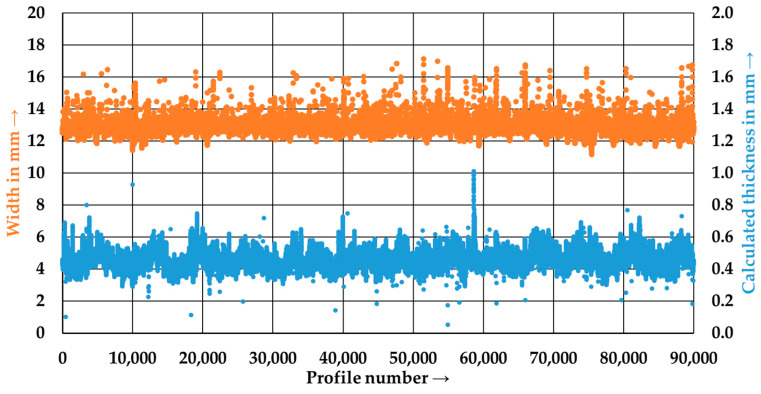
Width and calculated thickness of the tape P5_2 after the second pass through the tape manufacturing line.

Figure 8 shows the second pass of P5. The outliers in tape thickness were significantly reduced, and the mean value decreased to 0.46 ± 0.06 mm. The reduction in mean value can be attributed to improved tape consolidation.

**Figure 9:**
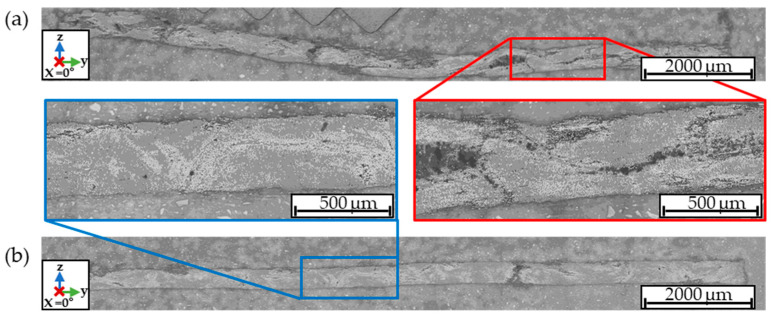
(**a**) Micrograph of P5_1; (**b**) micrograph of P5_2.

In order to compare the consolidation of the first and second pass of the P5 tape, polished cut images perpendicular to the process direction were prepared and are shown in Figure 9a,b. Furthermore, the tape width decreased to 12.82 ± 0.29 mm in the second pass. The decrease in tape width may be caused by fewer dry fibers at the tape edge due to better consolidation. Another possible reason is the movement of the tape within the calendaring unit, causing the tape to impact the groove of the consolidation roller at an angle during entry, leading to the folding and incomplete filling of the groove during the consolidation process.

Table 3 summarizes the width and thickness of the manufactured tapes after the first and second passes. It is evident that the width, thickness, and their respective standard deviations decrease with the second pass. It can also be observed that a higher stretching factor results in a decrease in tape thickness.

### 3.3. Characterization of Staple Fiber Organic Sheets

#### 3.3.1. Determination of the Fiber Volume Content

**Figure 10:**
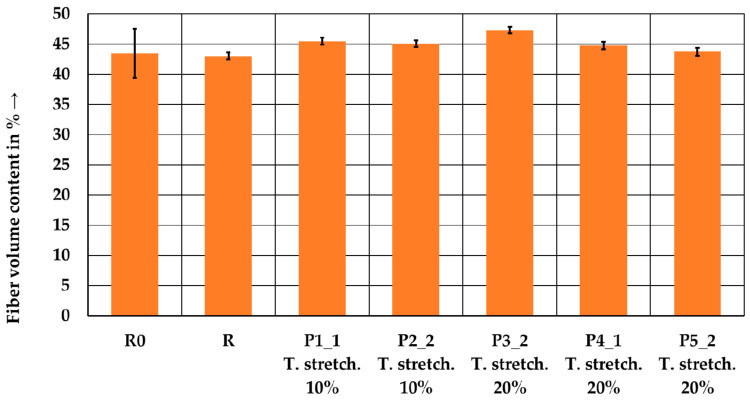
rCF fiber volume content for the yarns, reference laminate, and tape-laid laminates. Error bars represent the standard errors of the mean.

Figure 10 shows the results of the FVC measurements. The staple fiber yarns (R0) and the wound laminate exhibit lower values of 43.5% and 43.0%, respectively, compared to the tape-laid laminates. The tape-laid laminates have a fiber content of 43.7–47.3%. Already, the winding process seems to compensate for the large variation in the staple fiber yarn. Due to the use of 14 staple fiber yarns for one tape, the tape manufacturing process also results in significantly less scatter. Except for P5_2, all tape-laid laminates show a higher FVC compared to the wound laminate. It appears that the tape manufacturing process leads to a loss of matrix material.

#### 3.3.2. Determination of the Fiber Orientation

**Figure 11:**
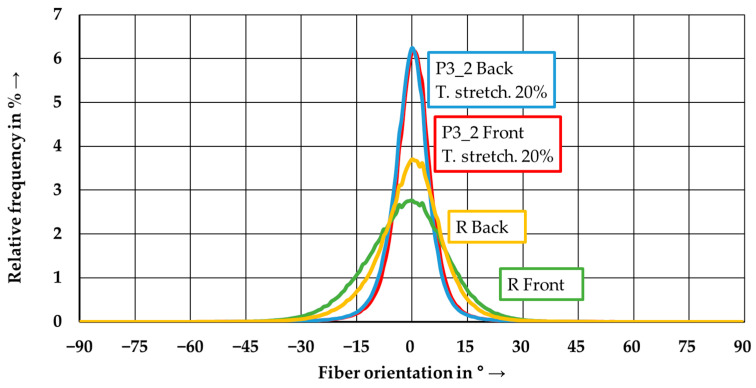
Fiber orientation distribution for the reference laminate R and the P3_2 tape-laid laminate (front and back side).

Figure 11 shows the fiber orientation of the wound reference laminate and, for comparison purposes, the tape-laid laminate from parameter set P3_2. The wound laminate had a lower fiber orientation than the tape-laid laminate. Furthermore, the differences between the front and the winding core facing the back side occurred. On the back side, the fibers were better aligned in the process direction. This can be attributed to the winding process, in which the first winding layer was precisely placed and fixed by subsequent layers, whereas the yarns were nested in the subsequent layers. In contrast, there was no difference between the front and back side, regarding the tape-laid laminates.

**Figure 12:**
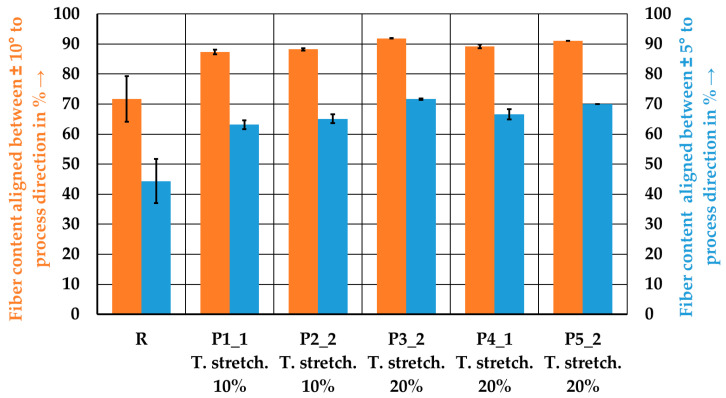
Fiber content aligned between ±10° and ±5° to the process direction for the reference laminate and the tape-laid laminates. Error bars represent the standard errors of the mean.

Figure 12 shows the averaged content of fibers oriented between an angle of ±10° and ±5° to the process direction from both the front and back sides. The tape manufacturing process leads to a homogenization of fiber orientation between the front and back sides. An increase in fiber content with an orientation of ±10° and ±5° to the process direction was observed compared to the wound reference laminate. The reference laminate has the lowest fiber orientation, with 44.4% between ±5° and 71.7% between ±10°. The laminate produced from tape P3_2 shows the best continuity in fiber orientation, with 71.6% at ±5° and 91.9% at ±10°. A higher stretching factor results in improved fiber orientation. The second pass without additional stretching in the tape manufacturing process (P2_2 and P5_2) leads to a slight improvement in fiber orientation compared to the single-stretched tapes (P1_1 and P4_1). This improvement can be attributed to better consolidation and a lower content of dry fibers at the tape edges. Only a slight difference in fiber orientation is detectable between the two-stage stretched tape P3_2 and the tape with the same total stretching and an additional pass without stretching, P5_2.

#### 3.3.3. Determination of the Mechanical Properties

**Figure 13:**
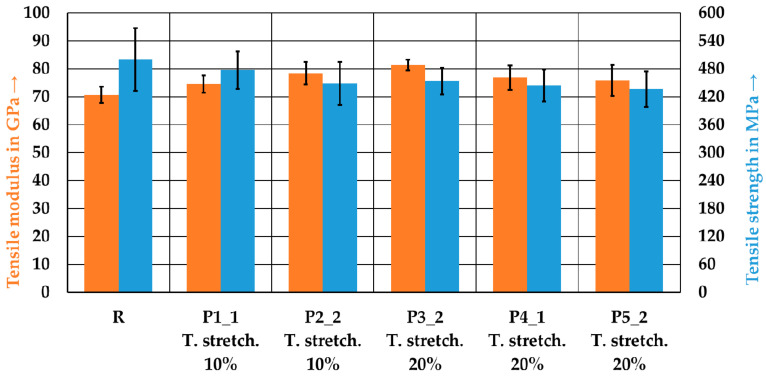
Tensile properties of the reference laminate and the tape-laid laminates. Error bars represent the standard errors of the mean.

Figure 13 shows the results of the tensile tests. The reference laminate exhibits the lowest tensile modulus but the highest tensile strength, with values of 70.7 GPa and 500.2 MPa, respectively. The laminate P5_2 shows the lowest strength at 436.3 MPa, whereas the laminate P3_2 exhibits the highest tensile modulus at 81.4 GPa. Although the fiber orientation for the P3_2 and P5_2 laminates was close to each other, they have differences in terms of the tensile modulus, as well as tensile strength. As elongation increases, the tensile strength decreases. However, these differences are only marginal. In contrast, increased stretching results in an increase in the tensile modulus.

**Figure 14:**
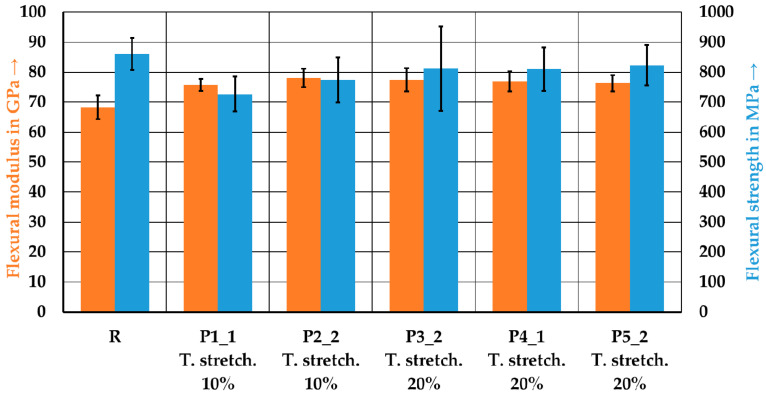
Flexural properties of the reference laminate and the tape-laid laminates. Error bars represent the standard errors of the mean.

Figure 14 shows the flexural strength and flexural modulus from the corresponding tests. The wound reference laminate exhibits the lowest flexural modulus but the highest flexural strength, with values of 68.2 ± 4.0 GPa and 860.9 ± 53.2 MPa, respectively. The laminate P1_1 has the lowest strength at 727.0 ± 58.0 MPa, whereas the laminate P2_2 exhibits the highest flexural modulus at 78.1 ± 3.1 GPa. Initially, the tape manufacturing process leads to a decrease in flexural strength, but with increased stretching, there is an increase in strength. The strength of the reference laminate was not achieved. The tape manufacturing process leads to an improvement in the flexural modulus, which could not be further improved by an additional stretching from 10% (P1_1 and P2_2) to 20% (P3_2, P4_1 and P5_2). The flexural strength could be improved by increasing the stretching.

#### 3.3.4. Determination of the Thermal Properties

**Figure 15:**
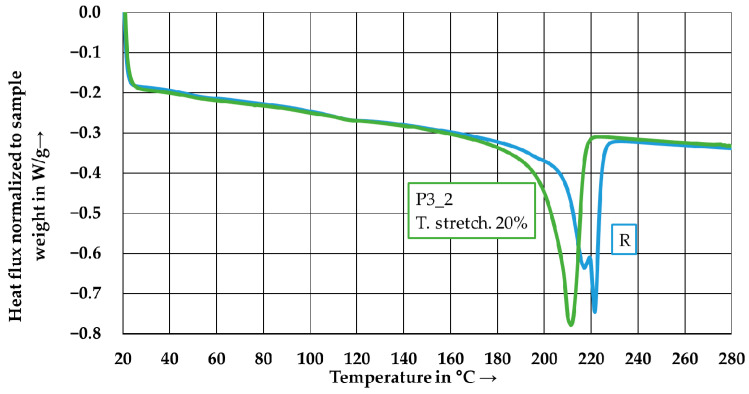
DSC-thermogram of the reference laminate and P3_2 tape-laid laminate.

Figure 15 shows the results of the DSC measurements conducted on the wound reference laminate and the tape-laid laminate P3_2. The first heating cycle is presented for both laminates, as the samples exhibit the same thermal history due to the identical autoclave cycle. A significant shift in the peak melting temperature to a lower value is observed in the tape-laid laminate P3_2. The peak melting temperature of P3_2 is 210.8 °C, whereas the wound laminate exhibits two peak melting temperatures at 217.2 °C and 221.7 °C, respectively. The tape-laid laminates P1_1, P2_2, P4_1, and P5_2 exhibit similar behavior to laminate P3, with peak melting temperatures varying between 208.8 °C and 212.4 °C. Therefore, the tape manufacturing process leads to an irreversible change in the matrix, which may be caused by degradation.

#### 3.3.5. Inspection of the Fracture Surface

**Figure 16:**
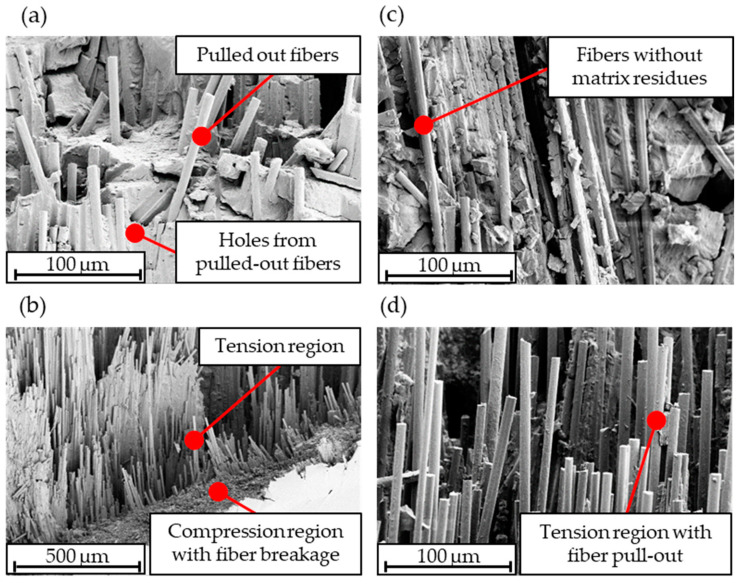
Scanning electron microscope images of the fractured surfaces from the samples of the reference laminate after the tensile test (**a**) and the flexural test (**b**), as well as the samples of laminate P3_2 after the tensile test (**c**) and the flexural test (**d**).

Figure 16 shows the fracture surfaces of the reference laminate and the tape-laid laminate P3_2 after the tensile test (a and c) and flexure test (b and d), respectively. The images of the tensile test specimens of both the reference laminate and laminate P3_2 reveal exposed fibers with a smooth surface without matrix residues. Additionally, impressions and holes from pulled-out fibers can be detected. For the flexural specimens, a clear transition can be observed between the region under compression and the region under tension (see Figure 16b). In the compression region, fiber breakage predominates, whereas, in the tension region, b and d, the fibers that are pulled out from the matrix are visible. This failure behavior in the tension-loaded areas indicates a lack of fiber-matrix adhesion, likely attributed to incompatible sizing.

## 4. Discussion

In Table 4, the determined properties normalized to a fiber volume content of 50% of the manufactured laminates, a reference rCF material, and a virgin UD-tape are summarized. This reference rCF material was selected because the tape was made of slivers consisting of 80 mm long rCF fibers within a PA6 matrix. In comparison to the reference rCF material (86%), a similar tensile modulus was determined, falling within the range of 80–84% of a virgin UD-tape. However, the tensile strength of the manufactured laminates (24–27%) is significantly below that of the reference rCF material (50%). Nevertheless, the manufacturing process led to improved flexural properties, with a higher flexural modulus (81–86%) and higher flexural strength (75–88%) compared to the reference rCF material (flexural modulus: 71%; flexural strength: 67%). While the sizing of the reference rCF material was tailored for a thermoplastic matrix, the sizing of the rCF used in this study was unknown. Through the examination of the fracture surfaces of the samples, a lack of fiber-matrix adhesion was detected. This insufficient fiber-matrix adhesion may be the cause of the decrease in tensile strength with increasing stretching. Improved fiber orientation in the samples leads to fewer form-fit connections due to poorly oriented fibers, and better-oriented fibers are simply pulled out from the matrix. This effect of increased fiber pull-out due to better orientation is likely to occur in the flexural tests as well but is overlaid by the influence of the compression region. In the compression region, fiber breakage predominantly occurs, which is influenced by fiber orientation. The increase in fiber volume content after the tape manufacturing process and the change in peak melting temperature compared to the reference laminate indicate that the processing of staple fiber yarns into tapes may have caused matrix degradation, which can also be responsible for poor fiber-matrix adhesion. This thermal degradation could also be a cause of the deterioration in flexural and tensile strength.

## 5. Conclusions

The presented process chain for processing staple fiber yarns is suitable for producing SF tapes, which can be further used in tape laying to manufacture load-bearing components. The conducted investigations confirmed the hypothesis that processing staple fiber yarns into SF tapes with an additional stretching step leads to an improvement in fiber orientation and homogenization of the fiber volume content. A second pass through the tape manufacturing line improves the consolidation of the tapes and reduces the presence of dry fibers at the tape edge, but a second pass through the process without further stretching results in only a slight improvement in fiber orientation. This improved fiber orientation results in an increase in both the flexural and tensile moduli. However, the tensile strength decreases during the tape manufacturing process, whereas the flexural strength initially decreases but then increases with increasing stretching and achieves values close to the reference laminate made of wound staple fiber yarns. In future investigations, reducing the temperature of the hot air blowers or increasing the speed of the system should be considered to decrease the energy input regarding the staple fiber yarns. The quantifiable determination of fiber-matrix adhesion through single-fiber pull-out tests, as well as analyzing the fiber length distribution in the staple fiber yarns before and after laminate production, should be the subject of future research.

## Figures and Tables

**Table 1:** Important physical properties of the Panex^®^ 35 carbon fibers.

Material Overview	SI
Tensile strength in MPa	4137
Tensile modulus in GPa	242
Elongation at break in%	1.5
Density in g/cm^3^	1.81
Fiber diameter in µm	7.2

**Table 2:** Process parameters in tape production.

Parameter Set	Temperature in °C Pass 1/ Pass 2	Speed v_1_ in m/min Pass 1/ Pass 2	Stretching Factor Pass 1/ Pass 2	Total Stretching Factor
P1_1 (Pass 1)	550	1.4	1.1	1.1
P2_1 (Pass 1) P2_2 (Pass 2)	550 550	1.4 1.1	1.1 1.0	1.1
P3_1 (Pass 1) P3_2 (Pass 2)	550 550	1.4 1.1	1.1 1.09	1.2
P4_1 (Pass 1)	550	1.4	1.2	1.2
P5_1 (Pass 1) P5_2 (Pass 2)	550 550	1.4 1.1	1.2 1.0	1.2

**Table 3:** Summary of tape width and thickness for the differently manufactured tapes. ± represents the standard errors of the mean.

Parameter Set	Width in mm	Thickness in mm
P1_1	12.88 ± 0.32	0.65 ± 0.14
P2_1	12.98 ± 0.41	0.70 ± 0.14
P2_2	12.86 ± 0.30	0.54 ± 0.07
P3_1	13.01 ± 0.31	0.67 ± 0.15
P3_2	12.87 ± 0.29	0.47 ± 0.07
P4_1	13.05 ± 0.40	0.60 ± 0.13
P5_1	13.05 ± 0.33	0.58 ± 0.11
P5_2	12.82 ± 0.29	0.46 ± 0.06

**Table 4:** Summary of the properties normalized to a fiber volume content of 50% for the manufactured laminates, a reference rCF material, and a virgin UD-tape. ± represents the standard errors of the mean.

Parameter Set	Tensile Modulus in GPa	Tensile Strength in MPa	Flexural Modulus in GPa	Flexural Strength in MPa
R	82.2 ± 3.4	581.6 ± 78.0	79.3 ± 4.7	1001.0 ± 61.9
P1_1 T. stretch. 10%	82.0 ± 3.3	524.5 ± 44.3	83.3 ± 2.2	798.9 ± 63.7
P2_2 T. stretch. 10%	87.0 ± 4.4	497.3 ± 50.8	86.6 ± 3.4	858.3 ± 82.9
P3_2 T. stretch. 20%	86.0 ± 2.0	479.7 ± 30.9	81.9 ± 4.1	858.1 ± 149.2
P4_1 T. stretch. 20%	85.8 ± 4.9	496.0 ± 38.5	85.8 ± 3.7	903.8 ± 81.5
P5_2 T. stretch. 20%	86.7 ± 6.4	499.1 ± 44.3	87.3 ± 3.0	941.9 ± 77.1
Reference rCF material [61]	89.0 ± 11.0	988.9 ± 33.9	72.2 ± 8.3	721.1 ± 51.4
Virgin UD-tape [62]	103.1	1969.1	101.1	1072.2

## Data Availability

The data presented in this study are openly available in Zenodo at 10.5281/zenodo.10148562 (accessed on 17 November 2023).
